# A Novel Dual-Payload ADC for the Treatment of HER2+ Breast and Colon Cancer

**DOI:** 10.3390/pharmaceutics15082020

**Published:** 2023-07-26

**Authors:** Candice Maria Mckertish, Veysel Kayser

**Affiliations:** Sydney School of Pharmacy, Faculty of Medicine and Health, The University of Sydney, Sydney, NSW 2006, Australia

**Keywords:** antibody drug conjugates, ADCs, antimitotic, microtubule polymerization, VcMMAE, SMCC-DM1, SK-BR-3, DLD-1, cytotoxic assay, trastuzumab, target specific

## Abstract

Antibody-drug conjugates (ADCs) have demonstrated a great therapeutic potential against cancer due to their target specificity and cytotoxicity. To exert a maximum therapeutic effect on cancerous cells, we have conjugated two different payloads to different amino acids, cysteines (cys) and lysines (lys), on trastuzumab, which is a humanised anti-HER2 monoclonal antibody. First, trastuzumab was conjugated with monomethyl auristatin E (MMAE), an antimitotic agent, through a cleavable linker (Val-Cit) to prepare ADC (Tmab-VcMMAE). Then, the ADC (Tmab-VcMMAE) was conjugated with a second antimitotic agent, Mertansine (DM1), via a non-cleavable linker Succinimidyl-4-(*N*-maleimidomethyl)cyclohexane-1-carboxylate (SMCC) to form a dual conjugate (Tmab-VcMMAE-SMCC-DM1). Our results indicated that the dual-payload conjugate, Tmab-VcMMAE-SMCC-DM1, had a synergistic and superior cytotoxic effect compared to trastuzumab alone. Ultimately employing a dual conjugation approach has the potential to overcome treatment-resistance and tumour recurrences and could pave the way to employ other payloads to construct dual (or multiple) payload complexes.

## 1. Introduction

Antibody-drug conjugates (ADCs) have generated great interest for the treatment of cancer. ADCs have demonstrated superior effects over standard chemotherapy agents for cancer due to their target specificity and efficacy [1]. Our study aimed at using a novel dual-payload ADC construct to target cancers that express the HER2+ antigen specifically in breast and colon cancer. The biomarker human epidermal growth factor receptor 2 (HER2) is expressed in some cancers, such as breast and colon cancer, but at different levels [2]. Gene amplification of the HER2 protein causes overexpression, resulting in higher-than-normal levels of the HER2 protein in cancerous cells [3].

Breast cancer is one of the most-diagnosed cancers globally according to the World Health Organization (WHO) [4,5]. Globally in 2020, breast cancer accounted for approximately more than 6.5% of mortalities and more than 11.5% of newly diagnosed cases of all cancer types [6], and colon cancer ranks third as the most common type of cancer and second as the most common cause of mortality worldwide compared to other cancer types [5]. Although a lot of progress has been made in recent years, targeted therapy is a rapidly evolving field and several ADCs have been approved.

As of December 2021, eleven ADCs have been approved by the US Food and Drug Administration (FDA), ten by the European Medicines Agency (EMA), and six by the Australian Therapeutic Goods Administration (TGA). Some of these ADCs have been developed for the treatment of various types of breast cancer; for example, T-DM1 (Kadcyla^®^: Genentech, South San Francisco, CA, USA), used for the treatment of metastatic breast cancer, targets HER2; sacituzumab govitecan (IMMU-132) (Trodelvy^®^: Gilead Sciences, Foster City, CA, USA), for triple-negative breast cancer, targets tumour-associated calcium signal transducer 2 (TROP-2); [fam]-trastuzumab deruxtecan (Enhertu^®^: Daiichi Sankyo, Chuo City, Tokyo, Japan/AstraZeneca, Wilmington, DE, USA), for the treatment of metastatic breast cancer and gastric cancer, also targets HER2 [7,8,9,10,11,12,13,14].

An ADC is comprised of a monoclonal antibody (mAb), a cytotoxic payload, and a linker that connects mAb and payload [15,16]. Upon conjugation of a mAb to a cytotoxic drug, the resulting ADC provides a synergistic effect compared to the mAb used alone or the payload alone [17,18,19]. For an ADC to successfully attain tumour cell death and cause apoptosis, it should have a high target binding specificity and affinity, a good internalization rate, low immunogenicity, a potent payload, and a stable linker [20]. An ADC binds to the antigen on cancer cells and enters the tumour cell through receptor-mediated endocytosis [7]. Upon entering the cell, the payload is released by a specific peptidase enzyme (i.e., cathepsin B) if a cleavable linker such as Valine-Citrulline (Val-Cit) is used for payload conjugation on a cysteine residue [1,7,21]. Payloads can also be conjugated to ADCs using non-cleavable linkers such as Succinimidyl-4-(*N*-maleimidomethyl)cyclohexane-1-carboxylate (SMCC) to a lys residues on a mAb. There are approximately 80 lys residues on a human IgG mAb, but only around 40 are accessible for bioconjugation, so conjugation to lys residues usually produce a somewhat heterogeneous population. In addition, most ADCs require proteolytic degradation of the mAb to release the payload after internalization [7,22,23,24,25,26,27].

The design and synthesis process of ADCs to produce the final product is a fairly complex and multistep procedure. For instance, if existing cys residues are used for payload conjugation after reducing the native disulfide bonds, the structure of the mAb may be compromised and further result in protein aggregation. Excessive protein aggregation is due to both the exposure of buried hydrophobic regions and structural disruption when conjugating via cys residues as well as to conjugating hydrophobic payloads on the surface of the protein. Aggregation of the mAb can cause undesirable immunogenic effects in the circulation and reduces the drug’s efficacy [28,29,30]. Hence, a thorough characterization is required in every step using many analytical techniques, including separation methods (e.g., size exclusion-high performance liquid chromatography (SE-HPLC), spectroscopy methods (e.g., ultraviolet visible spectroscopy (UV-Vis), mass spectrometry), and size determining methods (e.g., dynamic light scattering) [28,31,32,33,34,35].

When designing ADCs, other critical factors to keep in mind include toxicity levels or payloads and the accessibility of tumours. In terms of in vitro assays, the greater the drug load per mAb (8 drugs > 6 drugs > 4 drugs > 2 drugs), the better the half maximal inhibitory concentration (IC_50_) values. However, in vivo assays have revealed that a high drug load would most likely be too toxic, albeit this depends on the payload and its associated linker and has a higher clearance rate for some linker–payload constructs; hence, a low drug load might be favoured in some cases [7,36,37,38]. Moreover, the loading value is an essential parameter when determining the safety and efficacy of ADC [31]. Apart from this, the interstitial fluid pressures (IFP) within the tumour environment and the complexities of the extracellular matrix can hinder the movement of the therapeutic agent towards the tumour tissue. This can impact the penetration and retention of the target-specific therapeutic agent, leading to exposure to subtherapeutic doses [39,40,41]. The challenge lies in attaining a half-maximal inhibitory concentration at nanomolar levels or lesser while ensuring the tumour tissue is exposed to optimal doses of the therapeutic agent, in addition to overcoming the challenges displayed in the tumour environment including IFP and localization in the target tissue. It was shown that incorporation of the VcMMAE moiety to the Tmab structure increases the cytotoxic effect of the ADC due to its ability to successfully penetrate and localise in the target tissue [42].

This study incorporated the construction, characterization, and in vitro analysis of a novel dual-payload ADC conjugate employing two different small molecule drugs and linkers. Both payloads target microtubule disruption to construct Tmab-VcMMAE-SMCC-DM1 for an optimum effect. Each payload–linker complex was conjugated to different sites on trastuzumab. Evaluation with SE-HPLC of our ADC indicated that were no excessive aggregates present. This step is essential preceding in vitro studies with ADC. Further, we evaluated and demonstrated that a synergistic approach where each drug complements one another using two explicit anti-mitotic agents has the potential to address some of the above-mentioned limitations, including tumour penetration and retention of the target-specific therapeutic agent.

## 2. Materials and Methods

### 2.1. Materials

Tmab (Herceptin^®^) was generously donated by Genentech (San Francisco, CA, USA). Phosphate-buffered saline (PBS) was purchased from Astral Scientific (Gymea, NSW, Australia), SMCC-DM1 from Sapphire Bioscience (Redfern, NSW, Australia), EDTA and DTT were purchased from Sigma Aldrich (Macquarie Park, NSW, Australia), DTNB, Sodium bicarbonate, 50 kda Amicon Ultra-4 centrifugal unit from Merck (Bayswater, VIC, Australia), RPMI 1640 was purchased from Life Technologies (Carlsbad, CA, USA), VcMMAE, MMAE, and Mertansine/DM1 from Focus Bioscience (Murarrie, QLD, Australia), Incucyte^®^ cytotox green dye from Sartorius Australia, Dandenong South, VIC, Australia, EBS-9500-4633. SK-BR-3 cells were provided by Dr. Thomas Grewal (USyd) and DLD-1 cells were purchased from American Type Culture Collection (ATCC), local distributor In Vitro Technologies, CCL-221 (Noble Park North, VIC, Australia).

### 2.2. Synthesis of Tmab VcMMAE Conjugate

Tmab in formulation buffer was buffer exchanged with PBS 0.01 M, ethylenediaminetetraacetic acid (ETDA) 10 mM, 30 mM sodium bicarbonate at pH 8 to a final concentration of 3.8 mg/mL. Tmab was partially reduced with 10 mM of DTT for 90 min at 37 °C under stirring. The partially reduced Tmab was buffer exchanged with PBS 0.01 M and EDTA 1 mM with 50 kDa cut-off centrifugal filters, to remove the DTT. The linker–drug (VcMMAE) moiety was conjugated to free cys groups following partial reduction in Tmab. For this, VcMMAE was dissolved in DMSO at a concentration of 1 mM. This was added to the partially reduced Tmab at a ratio of 9.5:1 (VcMMAE:Tmab) under stirring for 1 h at 4 °C. The solution was quenched with 20-fold excess of cys in order to quench any unreacted or excess VcMMAE. xxx for a further 15 min under stirring at 4 °C. Unreacted VcMMAE and cys was removed through 50 kDa cut-off filters and stored in PBS at pH 7.4. A DAR of 2.83 was obtained through UV-Vis analysis.

### 2.3. Synthesis of the Tmab-SMCC-DM1 Conjugate

Following Tmab-VcMMAE conjugation, the linker–drug construct SMCC-DM1 was conjugated to Tmab-VcMMAE, to obtain a dual mAb-payload construct, Tmab-VcMMAE-SMCC-DM1. For this, the linker–drug complex SMCC-DM1 was dissolved in DMSO at a concentration of 1 mM and was added to the Tmab-VcMMAE conjugate at a ratio of 8:1 (SMCC-DM1:Tmab-VcMMAE ADC) and stirred for 4 h at 4 °C. Unreacted SMCC-DM1 was removed by centrifugation using 50 kDa cut off centrifugal filters and buffer exchanged with PBS 0.01 M at pH 7.4 for storage. A DAR 5.25 was obtained through UV-Vis analysis, based on absorbance peaks at 252 nm and 280 nm.

### 2.4. UV-Vis Spectroscopy

UV-Vis absorbance was recorded over a wavelength of 200–500 nm using Shimadzu 2600 UV-Vis spectrophotometer (Chiyoda-ku, Kyoto, Japan). The data was normalised and plotted using GraphPad prism version 9. The Beer–Lambert law was used to calculate the concentrations.

### 2.5. DTNB-Quantification of Free Thiol Groups

After confirming the integrity of the full-size antibody, free thiol groups per antibody in the sample were quantified using 5,5′-disthiobis 2-nitrobenzoic acid (DTNB) at an absorbance of 412 nm. DTNB reacts with a free sulfhydryl group to yield a mixed disulfide and 2-nitro-5-thiobenzoic acid (TNB), resulting in a yellow colour. Ellman’s reagent in reaction buffer was used as a blank.

The extinction coefficient used was $\varepsilon_{412}=1.4\times 10^{5}\;M^{-1}{\mathrm{cm}}^{-1}$.

### 2.6. Determining DAR (Drug-Antibody-Ratio)

The average drug-antibody ratio (DAR) was calculated based on the absorbance values at 248 nm and 280 nm. The extinction coefficients employed for MMAE were

$$\varepsilon_{248}=1.5\times 10^{3}\;M^{-1}{\mathrm{cm}}^{-1}\;\mathrm{and}\;\varepsilon_{280}=1.59\times 10^{4}\;M^{-1}{\mathrm{cm}}^{-1}$$

The formula employed:

$$DAR=\frac{\varepsilon_{Ab}^{248}-{H\varepsilon }_{Ab}^{280}}{{H\varepsilon }_{D}^{280}-\varepsilon_{D}^{248}}$$

where $H=A_{248}/A_{280}$, *Ab* = Tmab, *D* = MMAE, and the extinction coefficients utilised are listed in Table 1.

The formula employed:

$$DAR=\frac{\varepsilon_{Ab}^{252}-{H\varepsilon }_{Ab}^{280}}{{H\varepsilon }_{D}^{280}-\varepsilon_{D}^{252}}$$

where $H=A_{252}/A_{280}$, *Ab* = Tmab, *D* = DM1, and the extinction coefficients utilised are listed in Table 2.

### 2.7. SE-HPLC- Size Exclusion High Performance Liquid Chromatography

Following partial reduction, SE-HPLC was used with the in-line UV signal detector and was set at 280 nm to confirm the integrity of the full-size antibody and absence/presence of protein aggregates, using an Agilent 1200 Liquid chromatography system (Agilent Technologies, Santa Clara, CA, USA). The column used was TSKgel G3000SWXL 7.8 mm ID × 30 cm (TOSOH Bioscience, Tokyo, Japan). The mobile phase used was 0.15 M potassium phosphate buffer pH 6.5, flow rate was 0.5 mL/min, 10 µL was used as an injection volume, and measurements were performed in triplicate and repeated twice with two different vials.

### 2.8. Cell Maintenance and In Vitro Cytotoxic Assay

SK-BR-3 (high expressing HER2+ cells breast cancer cells) and DLD-1 (low expressing HER2+ cells) colorectal cancer cells were grown in RPMI media supplemented with 10% foetal bovine serum (FBS) and 1% of penicillin/streptomycin. Cells were maintained at 37 °C in a 5% CO_2_ humidified incubator.

Cells for SK-BR-3 and DLD-1 were seeded at 5 × 10^3^ cells per well in a 96 well plate. They were supplemented with RPMI media containing 10% FBS for 24 h and incubated at 37 °C in a 5% CO_2_ humidified incubator. After 24 h, the media was aspirated, the negative control was replenished with fresh RPMI media, and the cells were treated with fresh RPMI media containing Tmab, MMAE, DM1, and the double conjugate (Tmab-VcMMAE-SMCC-DM1) for 72 h. The samples were added at the corresponding concentrations in duplicates. Using the Incucyte^®^ ZOOM live-cell analysis system (Essen Bioscience, Ann Arbor, MI, USA). Images (at 10× magnification) were obtained at 2 h intervals from 4 different regions per well for 72 h after the addition of the treatment.

Generation of the curves was based on the cell confluence analysed using the Incucyte^®^ ZOOM integrated analysis software (v2016A). Growth rate (GR) inhibition metrics were employed to analyse the effect of the treatments. The data was fitted to a three-parameter dose response curve in which the GR values were plotted against treatment concentration. The GR_50_ was obtained based on the treatment concentration at GR = 50.

Colorimetric analysis was used to observe the cytotoxic effect of the samples using Incucyte^®^ cytotox green dye (Sartorius Australia, VIC, AU, EBS-9500-4633). The dye was initially diluted to 100 µM and further was diluted to 250 nM according to the manufacturer’s instructions (Sartorius Australia EBS-9500-4633) before addition to the RPMI media.

### 2.9. Statistical Analysis

**Figure 1:**
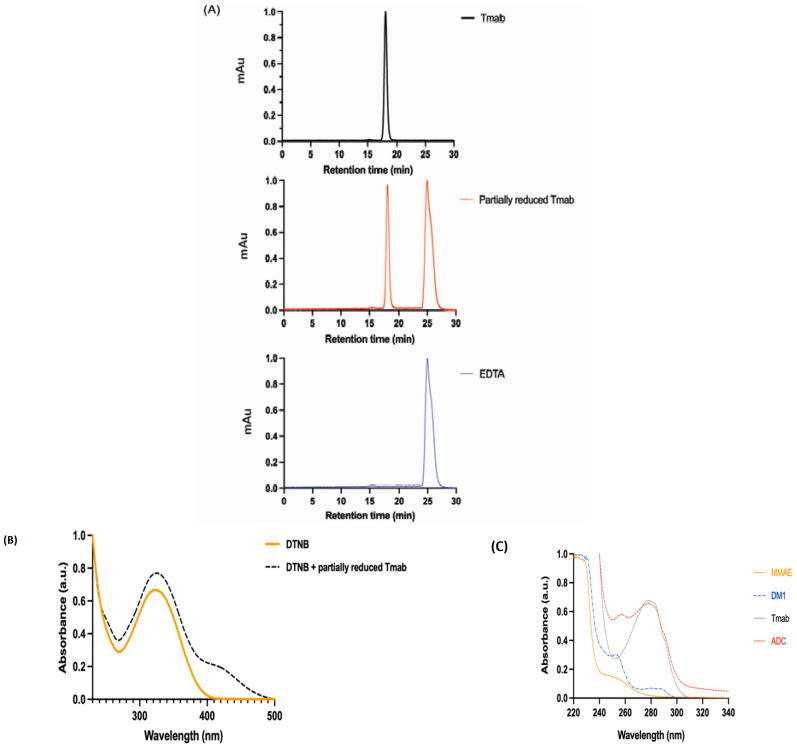
Characterization of ADC using HPLC and UV-Vis spectroscopy methods. (**A**) SE-HPLC of compares the intact Tmab and partially reduced Tmab, showing that the partially reduced Tmab remained intact after reduction with DTT. The EDTA peak was generated at 25 min. (**B**) Colorimetric analysis by Ellman’s reagent (DTNB). (**C**) The UV-Vis spectrum displays the peaks generated with the free drugs MMAE and DM1 at 248 nm and 252 nm, respectively. Tmab and the novel ADC displayed peaks at 280 nm.

**Figure 2:**
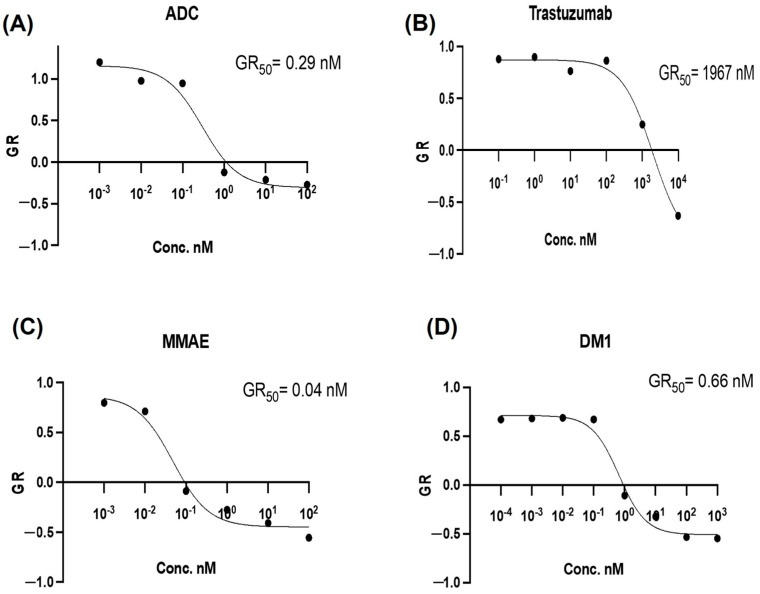
Growth rate inhibition curves for SK-BR-3 (*n* = 3): (**A**) Antibody–drug conjugate (ADC), (**B**) (trastuzumab) Tmab, (**C**) monomethyl auristatin E (MMAE), (**D**) mertansine (DM1).

**Figure 3:**
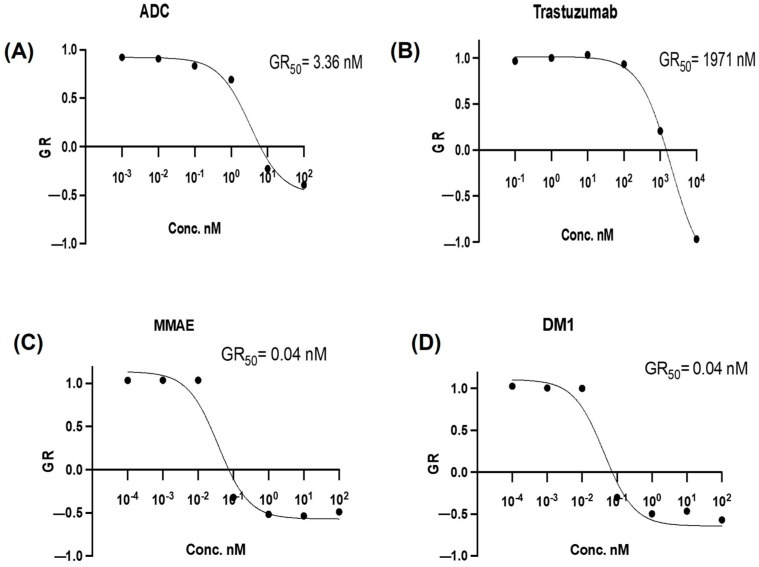
Growth rate inhibition curves for DLD–1 (*n* = 3): (**A**) antibody–drug conjugate (ADC), (**B**) (Trastuzumab) Tmab, (**C**) monomethyl auristatin E (MMAE), (**D**) mertansine (DM1).

**Figure 4:**
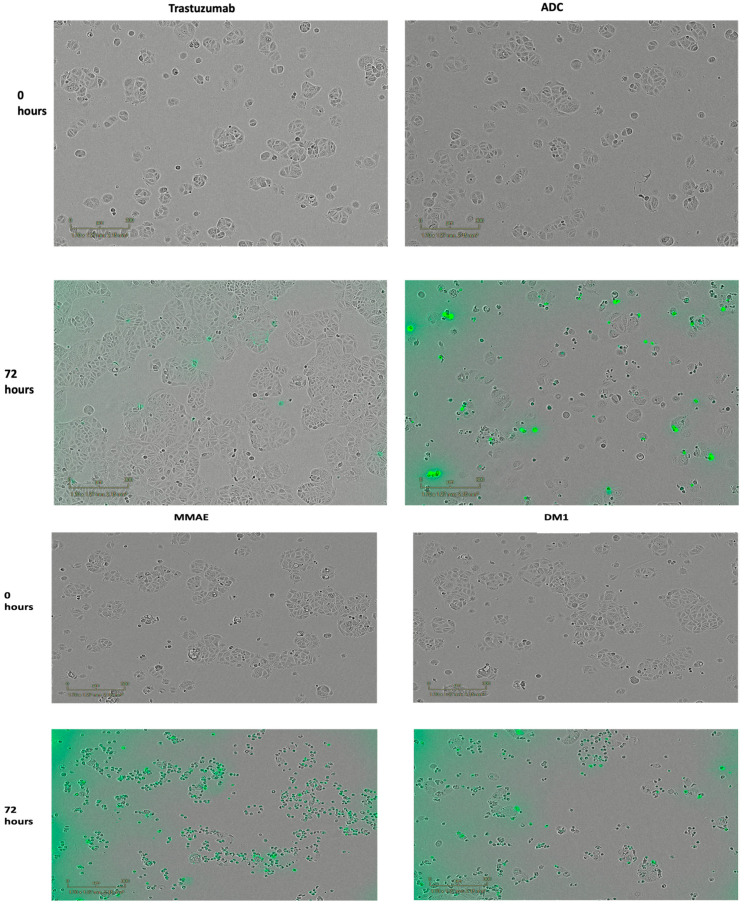
Cytotoxicity assays with SK-BR-3 cancer cells after 72 h with Incucyte^®^ cytotox green dye: free drugs MMAE and DM1, Trastuzumab and ADC. Images were obtained with Incucyte ZOOM Live-cell Analysis.

**Figure 5:**
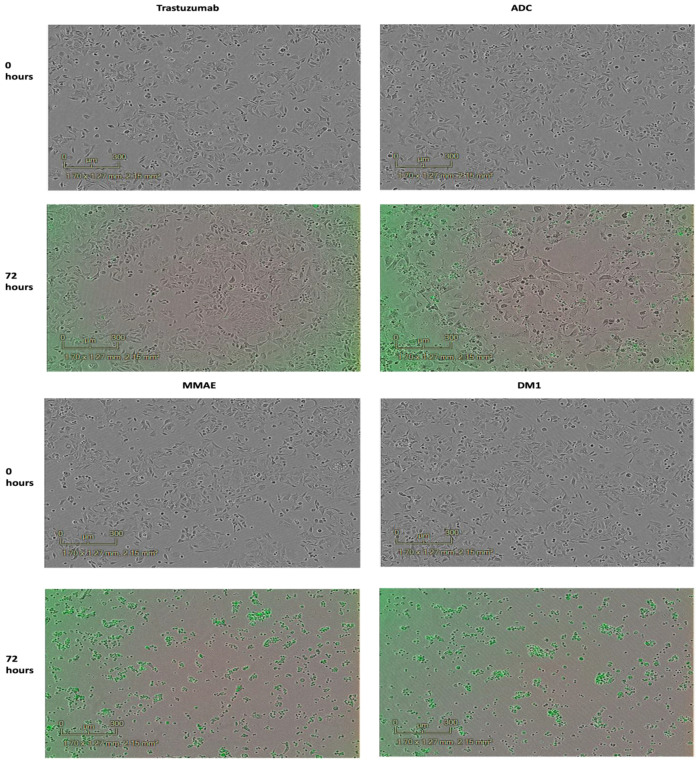
Cytotoxicity assays with DLD-1 cancer cells after 72 h with Incucyte^®^ cytotox green dye: free drugs MMAE and DM1, Trastuzumab and ADC. Images were obtained with incucyte ZOOM Live-cell Analysis.

**Figure A1:**
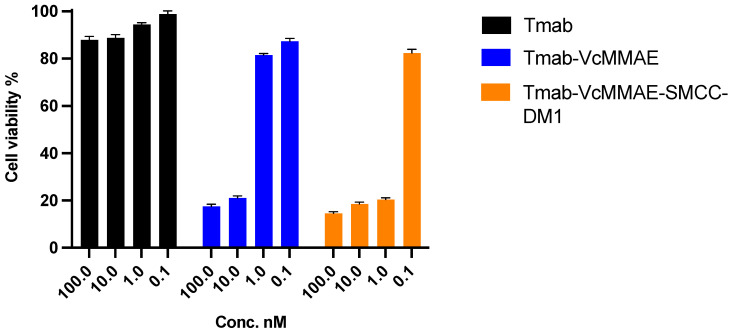
The cell viability graph indicates the percentage of viable cells following treatment. It demonstrated the superior synergistic effect of the dual conjugate (orange) compared to the single payload conjugate (blue) against the SK-BR-3 cell line. The figure indicates that even at a concentration of 1 nM, the dual conjugate (Tmab-VcMMAE-SMCC-DM1) was highly cytotoxic compared to the single payload conjugate (Tmab-VcMMAE).

The experiments were repeated three times (*n* = 3) and the cell viability data was analysed with a two-tailed, unpaired Student *t*-test and is presented as a mean ± standard deviation (*n* = 3) in Appendix A, Figure A3. The cell viability graph indicates the percentage of viable cells following treatment. The *p* < 0.05 was established as statistical significance. It highlights the superior synergistic effect of the dual conjugate compared to the single payload conjugate against the SK-BR-3 and DLD-1 cell line. Additionally, Figure A1 indicates that even at a concentration of 1 nM, the dual conjugate (Tmab-VcMMAE-SMCC-DM1) was highly cytotoxic compared to the single payload conjugate (Tmab-VcMMAE). The statistical analyses for Figure 1 is illustrated below and in Table 1 and Table 2. For Figure 2 and Figure 3, the corresponding statistical analysis can be found in Table 3 and Table 4 respectively. As for Figure 4 and Figure 5 the statistical analysis is represented in Appendix A.

## 3. Results

### 3.1. Design, Synthesis, and Characterization of the ADC (Tmab-VcMMAE-SMCC-DM1)

The mAb (Tmab) was characterised using SE-HPLC preceding linker–payload conjugation bonds, to ensure the absence of aggregates. Tmab was then partially reduced with the reducing agent (DTT) to break and expose interchain disulfide bonds. Figure 1A shows a sharp peak at 18 min, indicating that the Tmab and partially reduced Tmab were eluded at the same time intervals, confirming both proteins have similar elution profiles and no visible aggregates. The peak displayed at 25 min is a result of the presence of the EDTA (1 mM) buffer in which the mAb was stored.

Ellman’s reagent, also known as DTNB, is a colorimetric analytical technique that shows the presence of free thiol (SH) groups in the mAb sample after partial reduction. The absorption at 412 nm was used to calculate the number of free thiol groups in the sample based on the extinction coefficient $\varepsilon_{412}=1.42\times 10^{5}\;M^{-1}{\mathrm{cm}}^{-1}$. Figure 1B compares the absorbance of intact Tmab as a control and partially reduced Tmab in the presence of DTNB. Partially reduced Tmab trace is shifted in the y-axis different from the control Tmab, indicating the presence of a high number of free thiols on the protein, which was approximately 4.85 × 10^−5^ moles.

Payload conjugation was confirmed using UV-Vis as shown in Figure 1C, where the absorbance peak at 280 nm is due to Tmab, and peaks below 260 nm are due to payloads. The absorption spectrum of MMAE and DM1 contributes towards ADC absorbance, which allows the DAR estimation based on $A_{280}/A_{248}$ and $A_{280}/A_{252}$. The DAR obtained was 2.83 after conjugation with VcMMAE, calculated based on the absorption and extinction coefficients of the mAb and linker–drug complex in Table 1. The DAR obtained after conjugation with SMCC-DM1 was 5.25, calculated based on the absorption and extinction coefficients of the mAb and linker–drug complex in Table 2. DM1 had a higher drug loading compared to MMAE. The dual conjugated mAb (Tmab-VcMMAE-SMCC-DM1) displayed a strong absorbance at 280 nm compared to the spectrums of the free drugs displayed in Figure 1.

### 3.2. Cytotoxic Analysis of the ADC (Tmab-VcMMAE-SMCC-DM1)

The cytotoxic effect of the ADC was evaluated against the SK-BR-3 and DLD-1 cell lines, and results are shown in Figure 2 and Figure 3, respectively. Concentrations of the ADC are reported as molar concentrations based on the estimated DAR contributed by payloads, MMAE, or DM1. The cytotoxic effect of the dual conjugate in vitro assay was evaluated against HER2+ breast cancer and colon cancer cell lines. The half maximal growth rate (GR_50_) inhibitory concentration was derived from the GR metric measure. This metric measure was used to compare the GR_50_ value of the dual conjugate, MMAE, DM1, and Tmab. In SK-BR-3 cells, the ADC displayed 50% growth rate inhibition at a concentration of 0.29 nM (Figure 2), respectively. MMAE is a highly potent cytotoxin, demonstrating a potent effect against tumour cells [43] and, as a result, the cells exhibited a heightened sensitivity to MMAE compared to DM1. Evaluation of the GR metrics indicated that the SK-BR-3 cells were highly sensitive to the ADC.

In DLD-1 cells, the ADC displayed GR50 at a concentration of 3.36 nM (Figure 3A), whereas the unconjugated Tmab showed displayed a significantly higher GR50 at 1971 nM (Figure 3B). The DLD-1 cells were highly sensitive to the free drugs MMAE (Figure 3C) and DM1 (Figure 3D), both displaying a GR50 of 0.04 nM. Even though the DLD-1 cells were sensitive to the ADC, it displayed a superior cytotoxic effect against the SK-BR-3 cells attributable to its higher HER2 expression on tumour cells.

Moreover, in order to observe the cytotoxic effect of the ADC, cell images were captured using Incucyte ZOOM Live-cell Analysis prior to the addition of any sample and after a 72 h observation, as shown in Figure 4 and Figure 5. These images display the effect of the ADC on the cells, which was assessed based on morphological changes and a significant decline in the confluence of cells after 72 h. Figure 4 displays the images for the SK-BR-3 cell line. For the ADC, morphological changes to the cells were evident, as well as the number of proliferating cells significantly declined, supporting the growth rate inhibitory potential of the ADC. As expected, the confluence of cells was higher for the unconjugated Tmab and, conversely, the confluence of cells in the wells containing the free drug MMAE and DM1 was low displaying an inhibition in the growth rate. Figure 5 showcases images for the DLD-1 cell line, displaying comparable outcomes to those observed in Figure 4 when treated with the ADC, Tmab, MMAE, and DM1. The images captured for both cell lines indicate successful cell shrinkage and a reduction in the population of cells upon the addition of the ADC.

Furthermore, the results from the cytotoxic assay are summarised in Table 3 and Table 4, wherein the molar concentrations of the samples and the confidence intervals generated against SK-BR-3 and DLD-1 cells are outlined. The confidence intervals reinforce the reliability, certainty, accuracy, and precision of the GR_50_ value, and R^2^ values in Table 3 and Table 4, all of which are above 90%. This further amplifies the accuracy of the graphs generated in Figure 2 and Figure 3. Overall, in comparison with the control (Tmab), the ADC had a significantly lower GR_50_ against both the breast and colon cancer cell lines.

**Table 3:** GR_50_ values with confidence intervals (CI) obtained from the curves based on the cytotoxic effects of the samples on SK-BR-3 breast cancer cells in Figure 2.

Sample	GR_50_ (nM)	GR_50_ 95%CI *	R^2^
MMAE	0.04	0.002634 to 0.1642	0.9827
DM1	0.66	0.2991 to 1.463	0.9834
Dual payload	0.29	0.06723 to 1.288	0.9698
Tmab	1967	854.6 to 6091	0.9908

* CI: confidence intervals, GR_50_: concentration at which GR = 0.5.

**Table 4:** GR_50_ values with confidence intervals (CI) obtained from the curves based on the cytotoxic effects of the samples on DLD-1 colorectal cancer cells in Figure 3.

Sample	GR_50_ (nM)	GR_50_ 95%CI *	R^2^
MMAE	0.04	0.01167 to 0.1203	0.9657
DM1	0.04	0.01243 to 0.1162	0.9693
Dual payload	3.36	1.320 to 8.404	0.9879
Tmab	1971	1406 to 2864	0.9986

* CI: confidence intervals, GR_50_: concentration at which GR = 0.5.

## 4. Discussion

**Figure A2:**
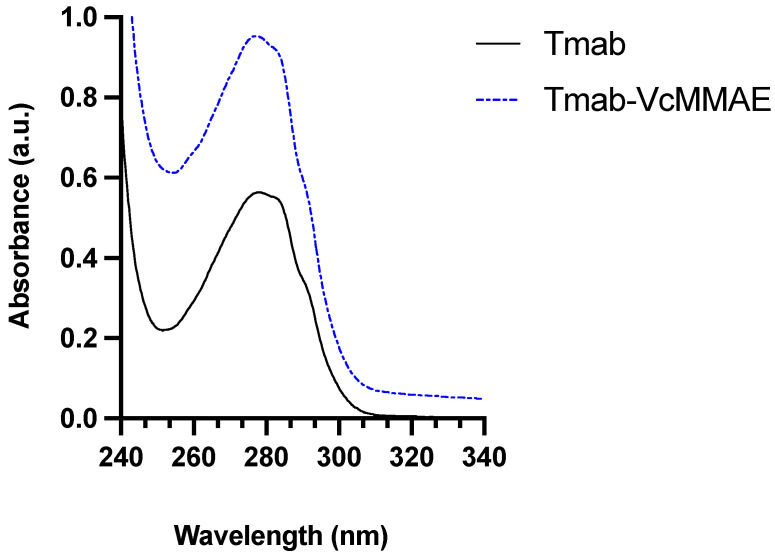
Characterization of the single payload conjugate (blue) and Tmab (black), demonstrating peaks at wavelength of 280 nm. The shift in the *y*-axis of the blue curve confirms payload conjugation.

**Figure A3:**
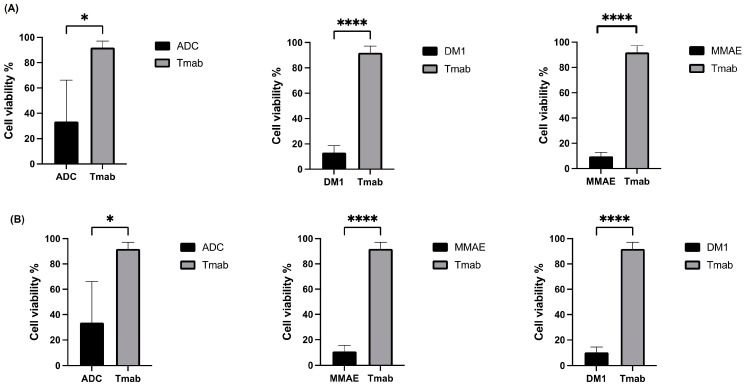
The experiments were repeated three times (*n* = 3) over 72 h, and the statistical analysis performed on the cell viability data is presented. (**A**) SK-BR-3 cells viability with the ADC, Trastuzumab (Tmab), Mertansine (DM1) and Monomethyl auristatin E (MMAE), (**B**) DLD-1 cell viability with the ADC, Trastuzumab (Tmab), Mertansine (DM1) and Monomethyl auristatin E (MMAE). The data is reported as a mean ± standard deviation * *p* < 0.05, **** *p* < 0.0001 (Student *t*-test) to represent the statistical significance.

ADCs have great potential in tackling many types of cancers and have gained wide use in recent years. For example, Trastuzumab emtansine (Kadcyla^®^, Genentech) was approved by the FDA, EU, and TGA in 2013 for the treatment of HER2+ metastatic breast cancer [8,9,44]. The cytotoxic payload DM1 is attached to the antibody via a nonreducible thioether linker 4-(*N*-maleimidomethyl)cyclohexane-1-carboxylate (MCC) [16,45]. However, there is always a concern about resistance demonstrated by the second-generation ADCs such as Trastuzumab emtansine (T-DM1) in advanced stages of the disease [46,47,48]. Our dual-payload modality has the potential to attain complete cell death and apoptosis through the attachment of SMCC-DM1 and VcMMAE. This means that the payloads can have a synergistic effect at the tumour site, and can exert a better therapeutic effect at a reduced concentration compared to a single payload conjugate. Although the two payloads target microtubules, their mechanism of action and binding sites are distinct. The MMAE known as an auristatin binds to the β-tubulin subunit of the microtubules, disrupting the assembly of microtubules by interfering with the alignment and organization of the tubulin subunits [49]. On the other hand, DM1, a maytansine derivative, caps and binds to the plus end of the growing microtubules, hindering the formation of mature microtubules and preventing the association of the tubulin subunits [49]. The synergistic potential and characterization of the dual-payload conjugate compared to a single payload conjugate is illustrated in Figure A1 and Figure A2, respectively. These results indicate that the use of two payloads that target microtubules but with a distinct mechanism of action can potentially overcome the treatment resistance and seed tumour recurrences and give rise to the potential for dual-payload conjugation for next-generation ADCs. In addition, ADCs are target-specific and, according to a study investigating the efficacy of an ADC comprised of trastuzumab and auristatin payloads against breast tumours, there was no evident toxicity observed against the HER2 negative cell line, HEK293, used in the study [50].

Both antimitotic agents employed in this study have a profound cytotoxic effect. They have been specifically conjugated to Tmab at DARs of 2.83:5.25 VcMMAE/SMCC-DM1, respectively, and characterised via UV-Vis spectroscopic analysis (Figure 1C). Against the SK-BR-3 cell line, the GR50 value of the dual conjugate was 0.29 nM, Tmab was 1967 nM, MMAE was 0.04 nM and DM1 was 0.66 nM after 72 h of incubation (Figure 2). However, against the DLD-1 cell line, the GR50 value of the dual conjugate was 3.36 nM, Tmab was 1971 nM, MMAE was 0.04 nM and DM1 was 0.04 nM after 72 h of incubation (Figure 3). Compared to the SK-BR-3 cell line, the GR50 value against the DLD-1 cells was higher, since DLD-1 cells have a low HER2 antigen expression [51] and SK-BR-3 cells have a high HER2 expression [52].

The nanomolar values of MMAE and DM1 mean that as free drugs, they more readily enter the cell by passive diffusion through the cell membrane and then target the tubuin, but these free drugs are not target-specific. The cleavable Val-Cit linker is stable in circulation and overcomes the concern of the payload being released before it enters the tumour [53]. It is designed to release the payload after receptor-mediated endocytosis following cleavage of the linker by the enzyme cathepsin B [54]. The bystander effect is attributed to the hydrophobic properties of a payload allowing it to diffuse through the cell membrane as well as target nearby tumours, can be beneficial for cancers that have a low and heterogenous expression of the target antigen [7,53,55,56,57,58]. The payloads were conjugated at a ratio of 2.83:5.25 VcMMAE/SMCC-DM1, respectively, to optimise the ADC design and due to the demonstrated benefits of incorporating a protease cleavable linker, MC-Val-Cit-PAB, also known as VcMMAE, that has the potential to effectively overcome acquired T-DM1 resistance and increase the sensitivity of the cells, making it a valuable addition to the ADC design [42,50]. Non-cleavable linkers such as SMCC are also very stable in circulation and are less prone to off-target toxicities [57]. A narrow therapeutic window usually arises from excess toxicity [59]. As MMAE is highly toxic at high drug loading, it has a high clearance rate in vivo and a narrow therapeutic window [37,57,60]. Because of this, MMAE has been used at a lower drug loading. Hence, we have incorporated a low drug loading of MMAE and a higher drug loading of DM1.

The GR_50_ value declined significantly from 1967 nM to 0.29 nM upon conjugation of the antibody to both antimitotic agents, displayed in Figure 2A, compared to the Tmab (GR_50_ = 1967 nM), Figure 2B. Colorimetric analysis using the Incucyte^®^ cytotox green dye (Figure 4 and Figure 5) shows cell internalization of our conjugate against SK-BR-3 and DLD-1 cells, respectively. The images are based on 72 h of analysis. These findings highlight the ADC’s apoptotic potential in effectively destroying cancer cells. With a GR_50_ of 0.29 nM, the ADC exhibited superior potency compared to the control (Tmab), which had a GR_50_ of 1967 nM against the HER2+ SK-BR-3 cells (Table 3). Similarly, the ADC exhibited a higher potency with a GR_50_ of 3.36 nM, compared to Tmab which resulted in a GR_50_ of 1971 nM against DLD-1 cells (Table 4). Both EGFR and HER2 have a role in colon cancer metastasis and development [61,62]. However, since the target antigen HER2 is expressed at a lower level in colon cancer compared to breast cancer [63], it can be attributed to the higher GR_50_ attained against DLD-1 cells compared to SK-BR-3 cells. Consequently, it can be concluded that in comparison to the antibody alone, which resulted in GR_50_ values of 1967 nM and 1971 nM against the SK-BR-3 and DLD-1 cells, respectively, the dual conjugate showed more potent antitumor activity resulting in GR_50_ values of 0.29 nM and 3.36 nM against the SK-BR-3 and DLD-1 cells, respectively.

Our data reaffirms that the dual conjugate approach, comprised of two different linker–payloads, both targeting microtubule disruption conjugated to different sites on the mAb (cys and lys), provided a synergistic effect against the cell lines, significantly reducing the growth rate of the breast and colon cancer cells. Through careful design, it is feasible to attach two distinct linker–payload complexes to separate sites on the antibody, without compromising the structural integrity of the mAb.

## 5. Conclusions

This study employed two explicit anti-mitotic chemical agents as payloads to create a dual-payload ADC construct and evaluated their synergistic approach. The ADC was characterised using separation methods and spectroscopy based analytical techniques. The payload MMAE was attached at a low drug loading and DM1 was attached at a higher drug loading due to their different toxicities. The payloads were conjugated to the mAb via different linkers that have demonstrated stability in the circulation, a necessary feature that is vital for successful payload delivery to the target site. The advantageous bystander effect that one of the linkers possesses means that it can be favourable for cancers, especially in DLD-1 cells that have a low HER2 expression. Our results indicate that the novel ADC conjugate maintained its affinity, and ability to penetrate the tumour, thereby exerting its cytotoxic effects and hence significantly reduced the growth rate of both tested cell lines, confirming our hypothesis that the incorporation of two cytotoxic agents could provide a synergistic effect. The superior cytotoxic effect of the new ADC construct against the SK-BR-3 cell line compared to the DLD-1 cell line can be attributed to the higher HER2 expression on SK-BR-3 cells compared to DLD-1. This concept paves the way for the feasibility of conjugating dual linker–payload moieties similar to this study or a manifold of linker–payload moieties to various mAbs depending on the target antigen, the tumour type, and the physical and chemical properties of the linker–payload moieties. This approach has the potential to overcome the burden of resistance as each linker–payload moiety employed can either target both microtubule polymerization and DNA or just one to ensure complete cell death and apoptosis. Further studies will investigate the in vivo activity of the novel dual conjugate.

## Figures and Tables

**Table 1:** Extinction coefficients of Tmab and MMAE for DAR based on UV-Vis spectroscopy.

Sample	$\varepsilon_{248}$	$\varepsilon_{280}$
Tmab	7.75 × 104	2.25 × 105
MMAE	1.50 × 103	1.59 × 104

**Table 2:** Extinction coefficients of Tmab and DM1 for DAR based on UV-Vis spectroscopy.

Sample	$\varepsilon_{252}$ (M^−1^cm^−1^)	$\varepsilon_{280}$ (M^−1^cm^−1^)
Tmab	76,565	2.25 × 10^5^
DM1	26,790	5700

## Data Availability

Not applicable.

## Abbreviations

ADC	Antibody-drug conjugates
ATCC	American Type Culture Collection
DAR	Drug-antibody-ratio
DM1	Mertansine
DTNB	5,5′-disthiobis 2-nitrobenzoic acid
DTT	Dithiothreitol
EDTA	Ethylenediaminetetraacetic acid
EMA	European Medicines Agency
FDA	Food and Drug Administration
GR_50_	Concentration at which growth rate = 0.5
GRI	Growth rate inhibition
HER2	Human epidermal growth factor receptor 2
IFP	Interstitial fluid pressure
mAb	Monoclonal antibody
MC	Maleimidocaproyl
MCC	4-(*N*-maleimidomethyl)cyclohexane-1-carboxylate
MMAE	Monomethyl auristatin E
nM	Nanomoles
PBS	Phosphate buffered saline
SE-HPLC	Size exclusion- high performance liquid chromatography
SMCC	Succinimidyl-4-(*N*-maleimidomethyl)cyclohexane-1-carboxylate
SMCC-DM1	Succinimidyl-4-(*N*-maleimidomethyl)cyclohexane-1-carboxylate Mertansine
Tmab	Trastuzumab
T-DM1	Trastuzumab emtansine
TNB	2-nitro-5-thiobenzoic acid
TROP-2	Tumour-associated calcium signal transducer 2
UV-Vis	Ultraviolet (UV)-visible spectroscopy
Val-Cit	Valine-Citrulline
VcMMAE	Valine-Citrulline Monomethyl auristatin E
WHO	World Health Organization
